# Differential ligand-signaling network of CCL19/CCL21-CCR7 system

**DOI:** 10.1093/database/bav106

**Published:** 2015-10-26

**Authors:** Rajesh Raju, Sachin Gadakh, Priyanka Gopal, Bijesh George, Jayshree Advani, Sowmya Soman, T. S. K. Prasad, Reshmi Girijadevi

**Affiliations:** ^1^Computational Biology Group, Cancer Research Program-9, Rajiv Gandhi Centre for Biotechnology, Thycaud, Poojappura, Thiruvanathapuram 690 014, Kerala, India and; ^2^Institute of Bioinformatics, Discoverer, International Technology Park, Bangalore 560 066, Karnataka, India

## Abstract

Chemokine (C-C motif) receptor 7 (CCR7), a class A subtype G-Protein Coupled Receptor (GPCR), is involved in the migration, activation and survival of multiple cell types including dendritic cells, T cells, eosinophils, B cells, endothelial cells and different cancer cells. Together, CCR7 signaling system has been implicated in diverse biological processes such as lymph node homeostasis, T cell activation, immune tolerance, inflammatory response and cancer metastasis. CCL19 and CCL21, the two well-characterized CCR7 ligands, have been established to be differential in their signaling through CCR7 in multiple cell types. Although the differential ligand signaling through single receptor have been suggested for many receptors including GPCRs, there exists no resource or platform to analyse them globally. Here, first of its kind, we present the cell-type-specific differential signaling network of CCL19/CCL21-CCR7 system for effective visualization and differential analysis of chemokine/GPCR signaling.

**Database URL:** http:// www. netpath. org/ pathways? path_ id= NetPath_ 46.

## Introduction

CCL19 and CCL21, also known as EBI1-Ligand Chemokine/Macrophage Inflammatory Protein-3β (ELC/MIP-3β) and Secondary Lymphoid-tissue Chemokine (SLC), respectively, are the major chemokines predominantly expressed in secondary lymphoid tissues (1–3). Elevated expression of the chemokines CCL19 and CCL21 have been reported in diverse disease conditions such as atherosclerosis (4, 5), cancers, bone disorders (6, 7) and other inflammatory conditions such as asthma (8), HIV infection (9) and pneumonia (10). Structurally, CCL21 differ from CCL19 in that it has an extra 32 amino acid C-terminus of basic amino acids that may mediate the distinct binding of CCL21 to other molecules (11, 12). Although, CCL19 and/or CCL21 have also been reported to bind to cell surface receptors such as chemokine (C-C motif) receptor-like 2 (CCRL2) and atypical chemokine receptor 4 (ACKR4), their functional effects are identified to be mediated through the class-A subtype GPCR, Chemokine (C-C motif) receptor 7 (CCR7) (13–16). Facilitating the migration, activation and survival of multiple cell types including dendritic cells, T cells, eosinophils, B cells, endothelial cells and different cancer cells, CCR7 signaling system has been implicated in diverse biological processes such as lymph node homeostasis, T cell activation, immune tolerance, inflammatory response and cancer metastasis (17–19). Dendritic cell- and stromal cell-based intratumoral delivery of CCL21 for enhanced recruitment and activation of antigen presenting cells and T cells are being evaluated for lung cancer therapy (20, 21).

Zidar *et al*. (22) have reported the differential and selective activation of the members of the G-protein-coupled receptor kinase (GRK)/beta-arrestin system of CCR7 by its two ligands, CCL19 and CCL21, with no substantial bias in the inhibition of adenylate cyclase by the CCR7-Gα_i/o_ system in HEK293 cells. Recently, Corbisier *et al*. (23) have reported based on bioluminescence resonance energy transfer assays that there is a ligand bias in the activation of multiple Gα_i/o_ isoforms of CCR7 in HEK293 cells. Subsequently, a large number of molecules and their involvement in mediating diverse biological processes of CCR7 have been analysed and reported under stimulation with either or both of these ligands in multiple cell types. With no further ligands currently established for CCR7 and taking into account the differences in sequences and their differential existence in soluble form as discussed by Comerford *et al*. (24), it is intriguingly significant to analyse the differential signaling between CCL19 and CCL21 in multiple cell types. To analyse the differential signaling of CCR7 ligands further, the experimentally proven molecular reactions induced by CCR7 ligands should be made available in computationally analysable formats such as BioPAX, SBML or GPML formats in a cell-type-specific manner (25–27).

To this end, we have compiled the first CCR7 signaling pathway and have made available the data in multiple exchangeable and analysable formats. The first of its kind, we have also have analysed and represented the experimentally reported molecular information available in CCR7 signaling to develop the cell-type-specific differential ligand-signaling network of CCR7.

## Curation of CCR7 Signaling Reactions

The genes and reactions reported to be induced by CCL19 or CCL21 through CCR7 were characterized into binary and complex protein–protein interactions, direct and indirect enzyme–substrate reactions, activation and inhibition status of proteins, protein translocations across multiple cellular compartments and genes differentially regulated at transcriptional and translational level, each of them following the criteria detailed before (28–30). The direct enzyme–substrate reactions should also be considered as protein–protein interactions. For each of the reactions, the specific cell types in which the reactions are studied were also documented. PathBuilder, an in-house pathway curation tool that facilitates quality check and thereby error-free curation was used to document all the reactions systematically (31).

## Identification of Differential-Ligand Induced Reactions

The different types of reactions aforementioned induced by similar dosage of CCL19 and/or CCL21 through CCR7 were analysed to identify reactions that were differentially induced by these ligands. We have currently characterized the differential-ligand induced reactions to a cell-type-specific manner. The reactions that were studied and identified to be induced by any one of the ligands without proof for the other ligand in a particular cell type are not considered as differential.

## Development of a Cell-Type-Specific Differential-Ligand Signaling Network of CCR7

### Molecular reactions reported in CCR7 signaling pathway

Manual analysis of the CCR7 signaling pathway reactions from 65 research articles out of a total of over 3000 articles that were screened resulted in the documentation of 24 protein–protein interaction events, 45 enzyme–substrate events, 13 protein translocation events. Together, we identified that 45 molecules were found to be activated and 9 proteins to be inhibited in CCR7 signaling for the dosage and time points reported in the published research articles. We have also documented 19 genes to be upregulated and 3 genes to be downregulated at the transcriptional and/or translational level.

### Cell-type-specific differential-ligand specific reactions

The analysis of the CCR7 signaling pathway reactions have resulted in the identification of 14 reactions to be differentially regulated in five cell types. For the other cell types, either CCL19 or CCL21 have been used to study the induced reactions (most of them belonged to this category) or that the differential signaling has not been identified and rather been reported as induced by both the ligands. The reactions that were identified to be differentially regulated are summarized in Table 1. In addition, the proteins reported to be activated in normal cells and multiple cancer cell types are provided in Table 2.

**Table 1.** The reactions reported to be differentially regulated by CCL19 or CCL21^a^

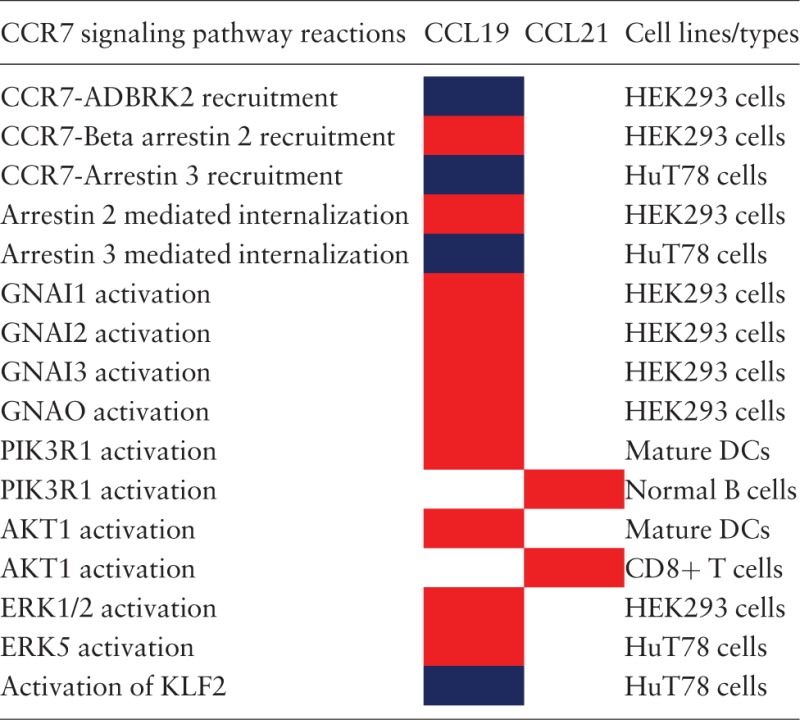

^a^The reactions differentially regulated by CCL19 or CCL21 in multiple cell types are distinguished by color codes. Red color box indicates that the corresponding ligand induces higher activation of the reaction than the other. Blue color box indicates that the reactions are identified to be induced solely by CCL19 and not by CCL21 in the specific cell lines studied.

**Table 2.** The proteins reported to be activated by CCL19 or CCL21 in normal and multiple cancer cell types^a^

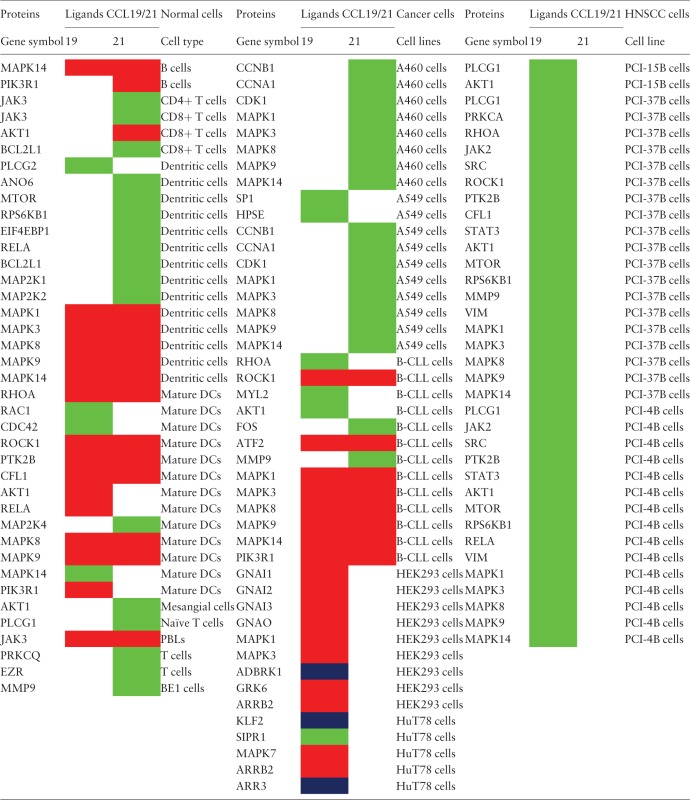

^a^The proteins regulated by CCL19 or CCL21 in multiple normal cells and cancer cell lines are distinguished by color codes. Red color box indicates that the corresponding ligand induces relatively higher activity of the protein than the other by virtue of increased activating post-translational modified forms with no significant differences in the protein amount. Blue color box indicates that the reactions are identified to be induced solely by CCL19 and not by CCL21 in the specific cell lines studied. The green color box indicates that the activity of the specific molecules is studied in particular cell types only for the corresponding ligand.

### Visualization of the CCR7 signaling pathway

**Figure 1.**
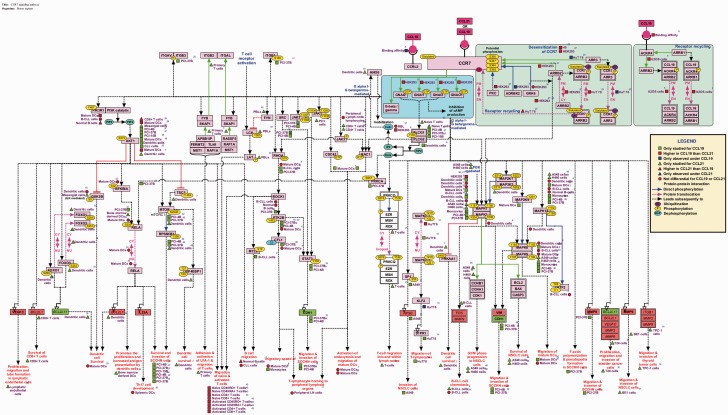
Cell-type-specific differential-ligand network map of CCL19/CCL21-CCR7 signaling. The network map of CCR7 signaling pathway reactions induced by its ligands CCL19 and CCL21 that are experimentally identified in multiple cell types are represented using PathVisio. The topology of the network is devised based on the mutants, inhibitors, activators and silencing approaches. The edges represent the reactions and nodes represent the molecules. Molecules italicized represent the genes transcriptionally regulated by CCR7 signaling with red indicating the upregulated genes and green, the downregulated genes. The edges are distinguished by colors to differentiate different types of reactions such as protein–protein interactions, Post-translational modifications (PTMs) and translocation reactions. The PTMs of each protein are represented with the site and residue of modification mapped to the corresponding RefSeq accessions. The differential signaling by CCL19 and CCL21 in multiple cell types are also represented using colored square (CCL19), triangle (CCL21) and circle (not differential for both CCL19 and CCL21) shapes. Blue represent the reactions to be unique to one of them but not induced by the other whereas red indicates higher for one and the green indicates studied only in one of them and not the other and hence not to be considered differential.

The pathway was manually represented using the visualization tool named ‘PathVisio’ (27). The topology was deduced based on the information obtained from assays using inhibitors/activators, silencing approaches, mutants and *in vitro* kinase assays. Specific reactions are denoted by edges connecting the nodes as represented in the legend in Figure 1. The differential signaling reactions in specific cell types have been represented using square, triangle and circle shapes distinguished by colors. Apart from the genes and their reactions, the biological processes or functions that were assayed to be mediated by the genes were also compiled with their specificity to cell types and differential-ligand stimulation whenever available.

### Data formats, availability and visualization

The CCR7 pathway data is free to the scientific community through the resource of signaling pathways, NetPath, at http://www.netpath.org/pathways?path_id=NetPath_46. The description to each molecular reactions and more information on specific molecules are available through the molecule pages in NetPath. The CCR7 signaling pathway data can be downloaded from NetPath in multiple data exchangeable and analysable formats such Proteomics Standards Initiative for Molecular Interaction (PSI-MI version 2.5) (32), Biological PAthway eXchange (BioPAX level 3) (25) and Systems Biology Markup Language (SBML level 2.1) (26). The gpml file can also be downloaded for visualization, analysis and editing from the NetSlim page in NetPath (http://www.netpath.org/netslim/ccr7_pathway.html). GPML format can be used for pathway enrichment analysis using GeneSpring (Agilent). The CCR7 signaling data provided in these formats may also be visualized and analysed using the software such as Cytoscape (33), GenMAPP, Visualization and layout services for BioPAX pathway models (VISIBIOweb) (34) and Chisio BioPAX Editor (ChiBE) (35). The pathway data will be updated periodically as more reactions become available.

## Conclusions

Signaling network maps are essential template for pathway analysis of high-throughput data. We have already developed over 40 signaling pathways facilitating this purpose and have made them available through NetPath. Here, we report not only the first chemokine signaling pathway map developed through the NetPath criteria but also the first ligand-specific and cell-type-specific signaling network induced differentially by two ligands of a single receptor, CCR7, the CCL19 and CCL21. We believe that this signaling pathway will serve as an efficient platform for the further analysis of the differential signaling between CCL19/CCL21 and also the reference for development of detailed maps for analysis of many more signaling pathways including GPCRs in the future.

## Funding

This study was supported by Department of Biotechnology (DBT), Government of India.

*Conflict of interest*. None declared.
